# ﻿A new genus of bamboo culm tarantula from Thailand (Araneae, Mygalomorphae, Theraphosidae)

**DOI:** 10.3897/zookeys.1080.76876

**Published:** 2022-01-04

**Authors:** Chaowalit Songsangchote, Zongtum Sippawat, Wuttikrai Khaikaew, Narin Chomphuphuang

**Affiliations:** 1 Faculty of Forestry, Kasetsart University, Bangkok, 10900, Thailand Kasetsart University Bangkok Thailand; 2 160 village no. 7, Mae Tho, Mueang Tak district, Tak, Thailand unaffiliated Tak Thailand; 3 5 village no. 8, Angthong sub-district, Chiang Kham district, Phayao, Thailand unaffiliated Phayao Thailand; 4 Department of Entomology and Plant Pathology, Faculty of Agriculture, Khon Kaen University, Khon Kaen, Thailand Khon Kaen University Khon Kaen Thailand

**Keywords:** Arboreal theraphosid, *Lampropelma*, *Melognathus*, *Omothymus*, *Phormingochilus*, *Taksinus*

## Abstract

Bamboo plays an important role in the animal world, including providing a nutritious food source, shelter, and habitat. Inside of bamboo culm, we discovered a new genus of tarantula, which we describe here as *Taksinus***gen. nov.** (♂♀). Specimens of this new tarantula were collected from Mae Tho, Mueang Tak district, Tak province, in Thailand, making it geographically distant from any other arboreal genera. Genital morphology was used to diagnose its genus, which is supported by distributional data, natural history, morphological characters, and photographic illustrations of the male and female. Diagnosis of the new genus was determined by distinguishing its different characters from those of other arboreal theraphosid spiders distributed throughout Southeast Asia. This tarantula’s specialization is that it lives in the stalks of the Asian bamboo *Gigantochloa* sp.

## ﻿Introduction

**Figure 1.**
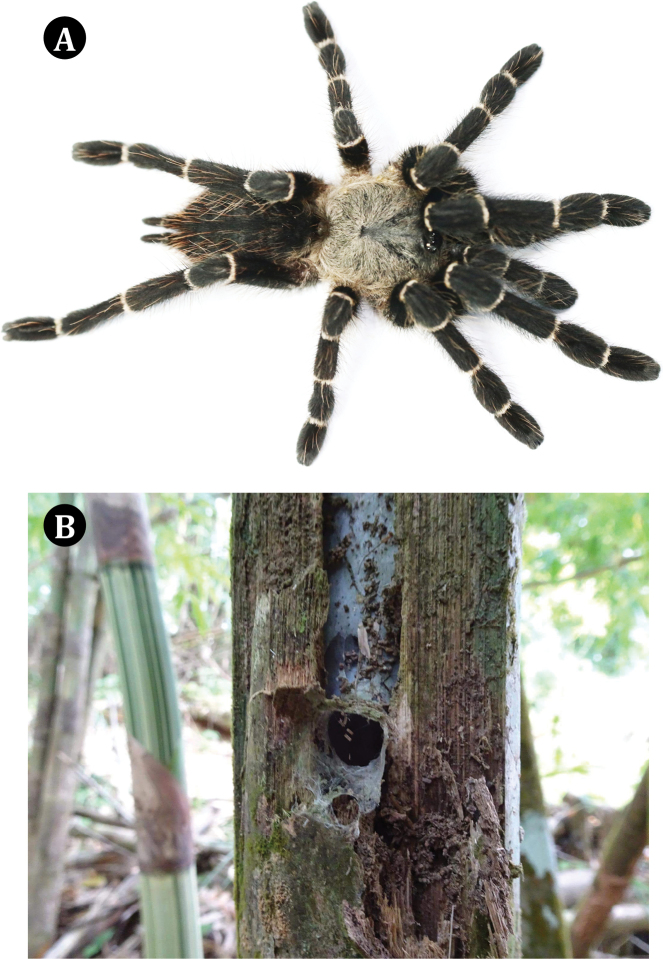
*Taksinusbambus* sp. nov. paratype ♀, TAK2 **A** dorsal view **B** habitat in bamboo culm.

**Figure 2.**
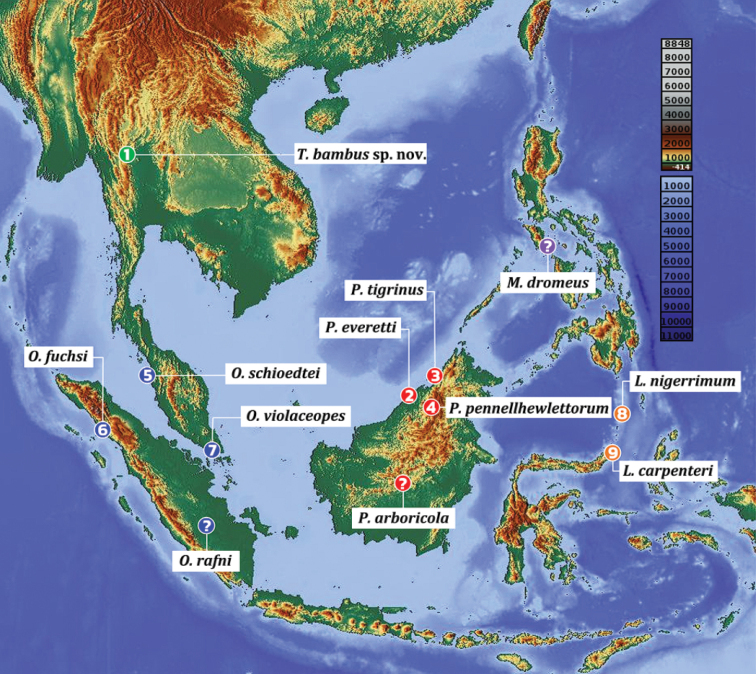
Distribution records of *Taksinusbambus* sp. nov. from Tak province, Thailand, and some arboreal Ornithoctoninae (Gabriel and Sherwood 2019), which demonstrate the separation of the genera by color. The topographic map of Southeast Asia and data were obtained from OpenStreetMap and OpenStreetMap Foundation OpenStreetMap (https://www.openstreetmap.org).

Theraphosidae Thorell, 1869, which are commonly known as tarantulas, comprises the most diverse family among Mygalomorphae Pocock, 1892, with over 1,000 species currently described (World Spider Catalog 2021). Asian tarantulas in the subfamily Ornithoctoninae Pocock, 1895, which are commonly known as earth tigers, were originally established by Pocock (1895). In this subfamily, four arboreal theraphosid genera have been recognized: *Omothymus* Thorell, 1891, *Lampropelma* Simon, 1892 *Phormingochilus* Pocock, 1895, and *Melognathus* Chamberlin, 1917. Tarantulas have not been observed to disperse aerially and thus have limited dispersal capabilities and habitat fidelities (Hendrixson et al. 2013). The type of localities and distribution of the Southeast Asian arboreal tarantula differ, as some species are restricted to a specific island; therefore, the spider’s location can be used to alongside morphology to diagnose species (Gabriel and Sherwood 2019). *Lampropelma* has been found in Indonesia (Sangihe Island and Sulawesi); *Omothymus* was reported in Malaysia, Singapore, and Sumatra; *Phormingochilus* currently contains four species, which are restricted to Borneo. The type locality of *Melognathus* is unclear. It was formerly reported as “East Indies? Philippines?” (Chamberlin 1917). In this study, we report a new genus, *Taksinus* gen. nov., discovered in northern Thailand, which is geographically distant from any previous record of the Asian arboreal species (Figs 1, 2). Historically, the taxonomy of arboreal Ornithoctoninae has been based primarily on unstable taxonomic characters, resulting in highly problematic identification characters being used before 2019. Pocock (1895) distinguished the genus *Phormingochilus* from *Omothymus* based on the anterior narrowness of the sternum. In the key of the Ornithoctoninae, Raven (1985) distinguished *Phormingochilus* based on the breadth of the ocular tubercle and the low caput. He defined *Lampropelma* from *Cyriopagopus* by the presence of brush setae on the retrolateral palpal femur. Smith (1994) proposed that the genus *Phormingochilus* could be distinguished by an absence of tibial apophyses and brush setae on the retrolateral face of the palpal femur, while exhibiting twin spermathecae. Smith and Jacobi (2015) reported that *Lampropelma*, *Omothymus*, and *Phormingochilus* share the following characteristics: a wide ocular tubercle, spermathecae with twin seminal receptacles, and low caput, whereas terrestrial tarantulas, such as *Cyriopagopus* have an extremely raised caput on the carapace. Recently, Gabriel and Sherwood (2019) revised the arboreal Ornithoctoninae and proposed stable taxonomic features, which clearly delineated the subfamily Ornithoctoninae, especially based on the male palpal bulb and tibial apophyses, which remain constant and relevant for characterizing arboreal species and genera. The comparative female leg measurements and geographic distribution can be used to further elucidate these taxa. Thailand currently has two genera of the tarantula subfamily Ornithoctoninae: *Ornithoctonus* Pocock, 1892 and *Cyriopagopus* Simon, 1887 (World Spider Catalog 2021). The newly recorded arboreal *Taksinus* gen. nov. was discovered in an extraordinary habitat, namely, bamboo culms with silken retreat tubes covering the stem cavity (Fig. 1A, B). The type localities of *Taksinus* gen. nov. and some Southeast Asian arboreal Ornithoctoninae are shown in Figure 2. We describe and provide illustrations of the body and copulatory organs, as well as information on the natural history and morphological characteristics that distinguish the new genus from other arboreal Ornithoctoninae.

### ﻿Materials and methods

Specimens were collected in Tak province, Thailand, on 21 July 2020. All tarantulas were preserved in 95% ethanol. Specimens were transferred to the Department of Entomology and Plant Pathology, Khon Kaen University, Khon Kaen, Thailand (ENTOKKU), for dissection and identification. The total body length, including cephalothorax and abdomen without spinnerets and appendage segments, were measured using digital vernier calipers. Diagnostic features and genitalia were photographed using a digital camera mounted to the phototube of a Nikon SMZ745T stereomicroscope, and the NIS-Elements D program was employed for measurement and counting morphology. Appendage measurements were made dorsally along the central axis of the measured structures from the left side and recorded in millimeters (mm). The length ratio of leg I–leg IV multiplied by 100 (von Wirth and Striffler 2005) was used to calculate the relation factor (RF), which was compared with data from Gabriel and Sherwood (2019) who proposed the difference between the total lengths of leg I and IV to show the distinct characteristics of *Phormingochilus*, *Lampropelma*, and *Omothymus*. The leg formula, with the leg length in decreasing order, is also provided. Unless otherwise noted, the color of the morphological character was observed in ethanol-preserved specimens. Female genitalia were dissected and cleared in 3 M aqueous KOH solution. Specimens were identified by comparing them to related species (Smith and Jacobi 2015; Gabriel and Sherwood 2019). Specimens are deposited in the Entomology Museum, Faculty of Agriculture, Khon Kaen University (**ENTOKKU**) in Khon Kaen, Thailand, and the Natural History Museum of the National Science Museum (**THNHM**) in Pathum Thani, Thailand. The following abbreviations are used in the text to describe characters: **AER** = anterior eye row; **AME** = anterior median eyes; **ALE** = anterior lateral eyes; **PER** = posterior eye row; **PME** = posterior median eyes; **PLE** = posterior lateral eyes, **MOA** = median ocular area, **PLS** = posterior lateral spinnerets; **PME** = posterior median eyes; **PMS** = posterior median spinnerets, **Fem** = femur, **Pat** = patella, **Tib** = tibia, **Met** = metatarsus, **Tar** = tarsus. The terminology for leg spines is based on Petrunkevitch (1925), with modifications proposed by Bertani (2001): **r** = retrolateral, **p** = prolateral, **d** = dorsal, and **v** = ventral. If all the spines in the apical part were apically positioned, the term “apical” would be used to refer to their position.

### ﻿Other material examined

*Omothymus* sp. 2 ♂ Surat Thani and Chumphon, Thailand.

*Omothymus* sp. 1 ♀ specimen was donated from an unknown locality.

*Cyriopagopusalbostriatus* (Simon, 1886) 2 ♀ Saraburi, Thailand.

*Cyriopagopusminax* (Thorell, 1897) 3 ♀ Chiang Mai, Thailand.

*Cyriopagopuslividus* (Smith, 1996) 1 ♂ Chanthaburi and 4 ♀ Trat, Thailand.

*Cyriopagopuslongipes* (von Wirth & Striffler, 2005) 1 ♂ and 5 ♀ Ubon Ratchathani, Thailand.

*Ornithoctonusaureotibialis* von Wirth & Striffler, 2005 2 ♀ Chumphon, 2 ♀ Ranong, and 2 ♀ Krabi, Thailand.

*Ornithoctonuscostalis* (Schmidt, 1998) 2 ♀ Phetchaburi, Thailand.

## ﻿Taxonomy

**Mygalomorphae Pocock, 1892**

**Theraphosidae Thorell, 1869**

**Ornithoctoninae Pocock, 1895**

**Included genera**: *Citharognathus*, *Cyriopagopus*, *Lampropelma*, *Melognathus*, *Ornithoctonus*, *Phormingochilus*, *Taksinus* gen. nov.

### ﻿ Taksinus

Taxon classificationAnimaliaAraneaeTheraphosidae

Songsangchote, Sippawat, Khaikaew & Chomphuphuang gen. nov.

2AA74850-A8ED-5C59-AF20-1172A240B263

http://zoobank.org/AB5E52BE-415F-415E-91AC-1155EA142B1D

#### Type species.

*Taksinusbambus* Songsangchote, Sippawat, Khaikaew & Chomphuphuang, 2021 from Tak, Thailand.

#### Diagnosis.

The characteristics of *Taksinus* gen. nov. that differ from *Ornithoctonus* and *Cyriopagopus* are: a low caput, a clypeus that is less than the width of the median ocular quadrangle (Fig. 6A), and spermathecae with twin seminal receptacles (Fig. 7E, F) (Raven 1985; von Wirth and Striffler 2005; Smith and Jacobi 2015). The new genus differs from *Citharognathus* by the lack of incrassate tibia and metatarsus IV. *Taksinus* gen. nov. differs from *Lampropelma* by the absence of a dense brush of hair on the retrolateral side of the femora of the front limbs (von Wirth and Striffler 2005) and males by lack of apical embolus swelling (Fig. 5A–E; see Gabriel and Sherwood 2019: 143, figs 17, 18). *Taksinus* gen. nov. can be distinguished from *Omothymus* by male palpal bulb with a gently curved embolus with rounded embolic apex (Fig. 5A–E) vs palpal bulb steep angle embolus and apex with a sharp point in *Omothymus* (Fig. 5F–J; see Gabriel and Sherwood 2019: 139, figs 1–5). *Taksinus* gen. nov. differs from *Phormingochilus* by the lack of a single megaspine on the inside of the male tibial apophyses (Fig. 4A, B; see Smith and Jacobi 2015: 41, fig. 38; Gabriel and Sherwood 2019: 142, figs 14–16), a short embolus compared to palpal bulb length (1:1) (Fig. 5A–E), and the geographic distribution of *Phormingochilus* currently restricted to Borneo.

#### Etymology.

The generic name was named Phraya Tak (governor of Tak province), which is in honor of Taksin the Great, king of the Thonburi Kingdom, in commemoration of his early career.

#### Description.

Carapace longer than wide, low caput. Fovea deep, straight (males) or slightly procurved (females). Clypeus short, less than width of median ocular quadrangle in males and females. Eight eyes arranged on tubercle, anterior eye row slightly procurved and the posterior row straight. Outer cheliceral on lower surface from margin with five slightly curved pad of plumose setae on the retrolateral chelicerae. Maxillae longer than wide with >155 cuspules (male) or 149–183 (females), two horizontal rows of 10–11 stout thorn-like spines on the lower half of prolateral maxillae (below suture) and one horizontal row of six stout thorn-like spines on the upper half of prolateral maxillae (above suture). Spines of varying lengths, with the longest being at the top of the series; combined to form a stridulating organ. Labium wider than long, with 75 cuspules (male) or 125 (females). Sternum longer than wide, with two pairs of ovoid sigillae; Posterior sigilla is significantly remote from the edge, middle sigilla is close to the margin, and anterior sigilla is indistinguishable. Legs: formula 1423 (males); ± Total lengths of legs I and IV = 0.48, 4123 (females) ± Total lengths of legs I and IV = 2.41–3.33, RF = 101 (males) or 90.6–93 (females). Scopulae distinct, thickly set on tarsus; ventral surface not divided. Tibial spur capped with multitude of thin, short black spines, with no single megaspine on the inside of the tibial apophyses. Palpal bulb is ellipsoid and partly concave, embolus short compared to palpal bulb length (1:1), moderately curved, rounded apex, with single retrolateral keel. Spermathecae have twin seminal receptacles, rounded tombstone receptacles, fused in the basal region.

#### Distribution.

Tak province, Thailand

### ﻿ Taksinus bambus

Taxon classificationAnimaliaAraneaeTheraphosidae

Songsangchote, Sippawat, Khaikaew & Chomphuphuang sp. nov.

B76D07C9-ABFC-5150-905A-49AD6B7075A7

http://zoobank.org/B377A56A-9162-412D-8AF9-462759CF8F4F

**Figure 3.**
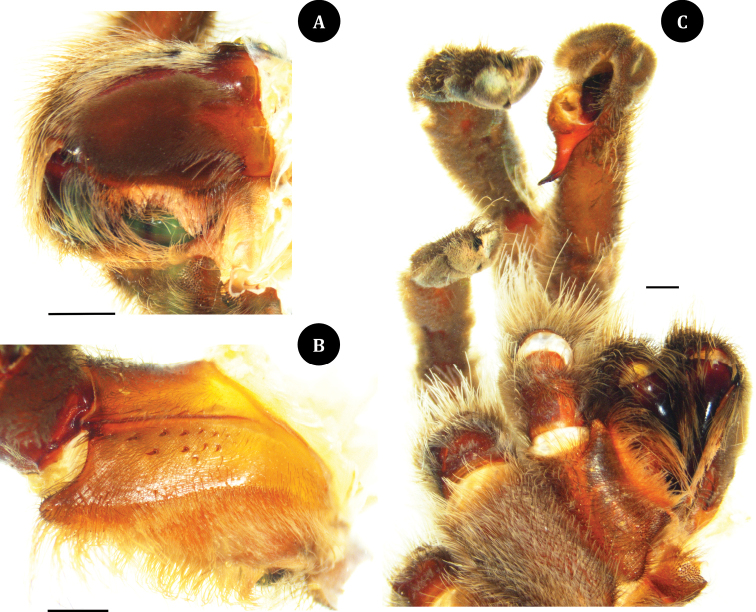
*Taksinusbambus* sp. nov. Holotype ♂ TAK1 **A** chelicerae, retrolateral view **B** maxilla, prolateral view **C** palpi, labium, maxilla, and coxae, ventral view. Scale bars: 1 mm.

**Figure 4.**
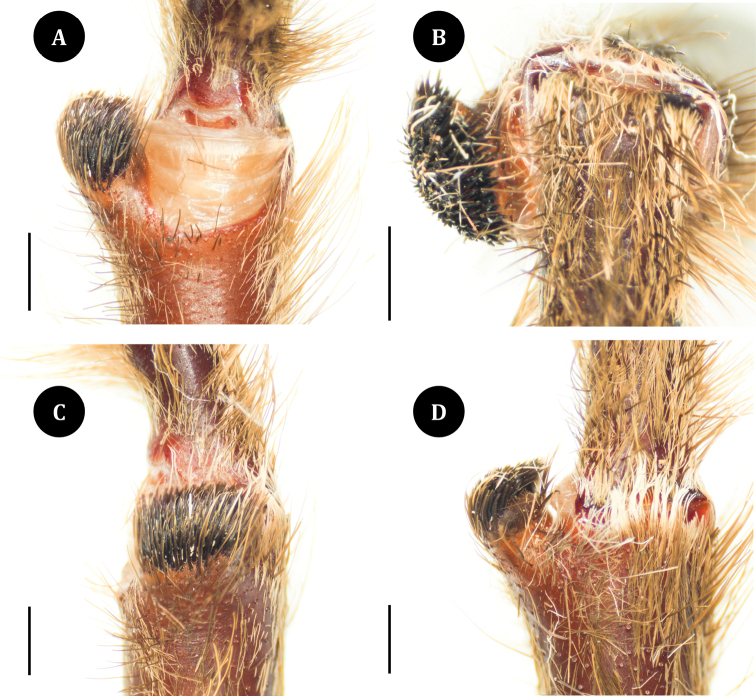
*Taksinusbambus* sp. nov. Holotype ♂ TAK1. Tibial apophysis **A** ventral view **B** apical view **C** prolateral view **D** dorsal view. Scale bars: 1 mm.

**Figures 5.**
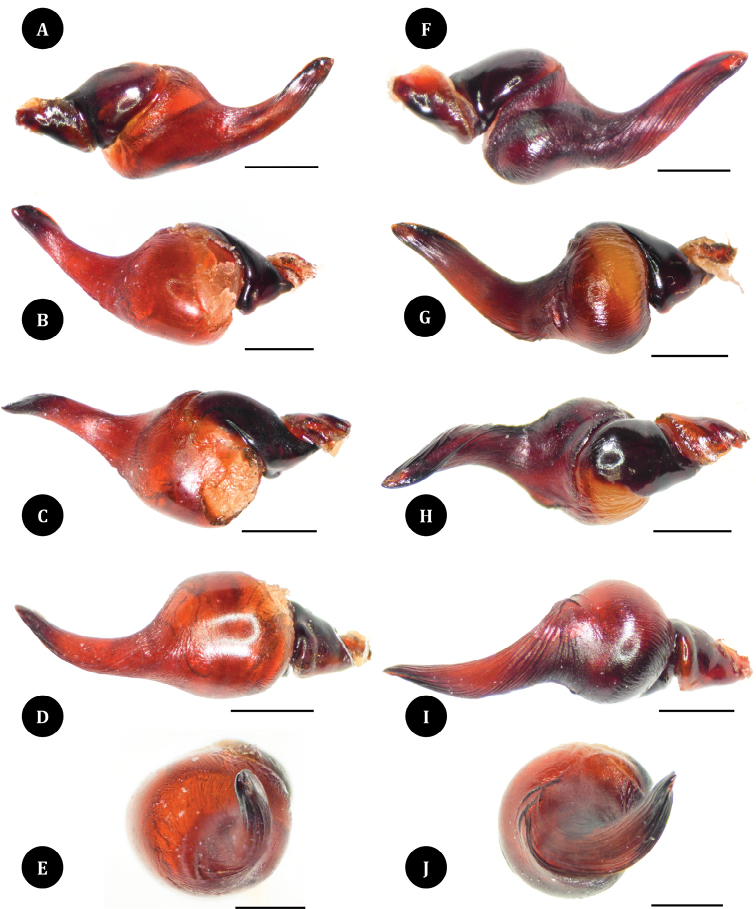
*Taksinusbambus* sp. nov. holotype ♂ TAK1 (**A–E**) and *Omothymus* sp. ♂ (**F–J**). Palpal bulb **A** prolateral view **B** retrolateral view **C** dorsal view **D** ventral view **E** apical view **F** prolateral view **G** retrolateral view **H** dorsal view **I** ventral view **J** apical view. Scale bars: 1 mm.

**Figure 6.**
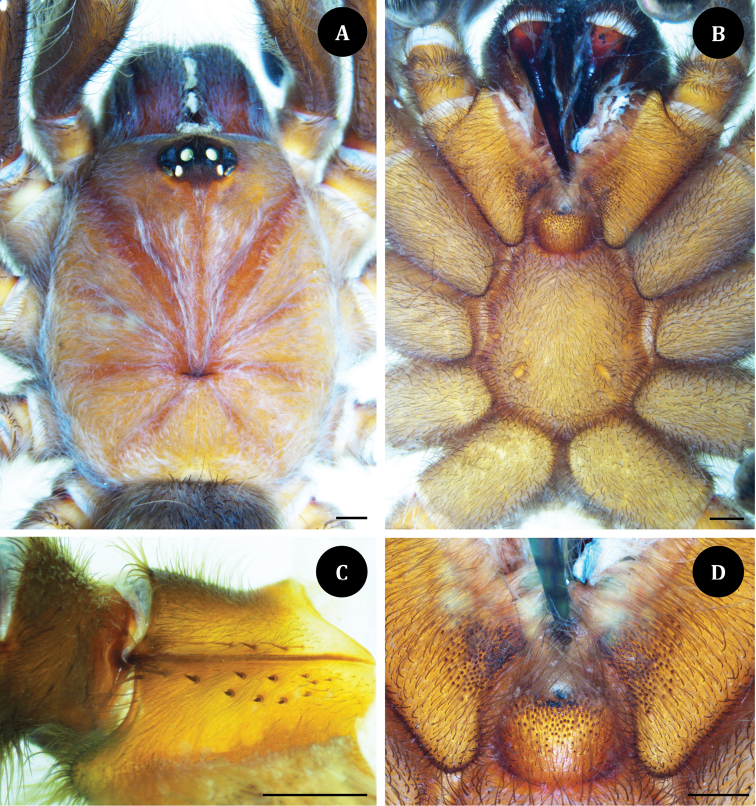
*Taksinusbambus* sp. nov. Paratype ♀ TAK3 (**A, B, D**) Paratype ♀ TAK2 (**C**). **A** carapace, dorsal view **B** sternum, labium, maxilla, and coxae, ventral view **C** maxilla, prolateral view **D** maxillae and labium with cuspules. Scale bars: 1 mm.

**Figures 7.**
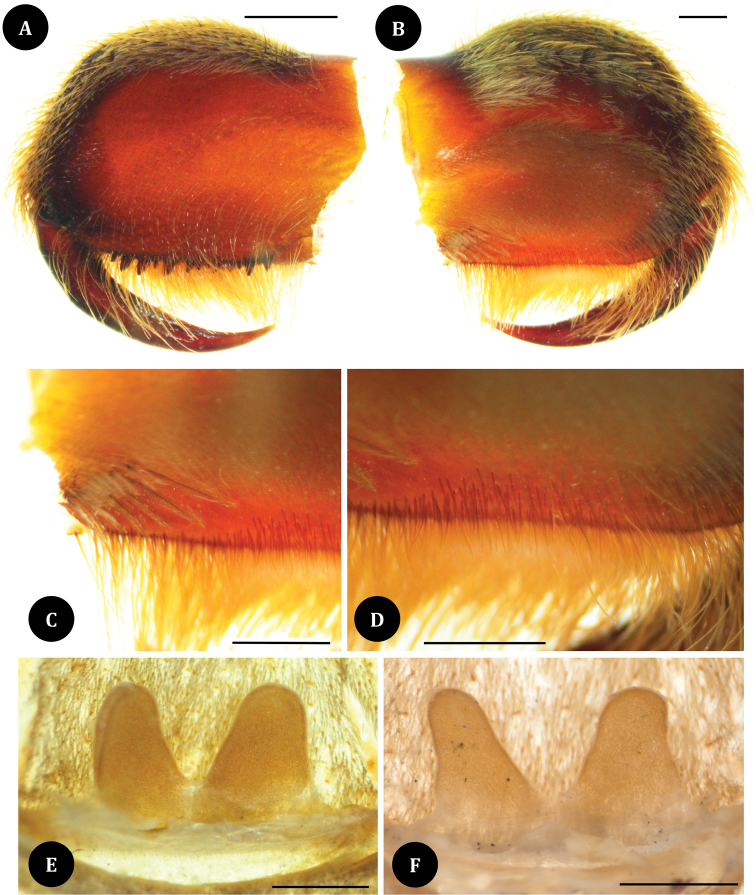
*Taksinusbambus* sp. nov. paratype ♀ TAK2 (**A–E**) paratype ♀ TAK3 (**F**). **A** chelicerae, prolateral view **B** chelicerae, retrolateral view **C** plumose hairs outer chelicerae, retrolateral view **D** chelicerae strikers, retrolateral view **E** spermathecae, dorsal view **F** spermathecae, dorsal view. Scale bars: 1 mm.

**Figures 8.**
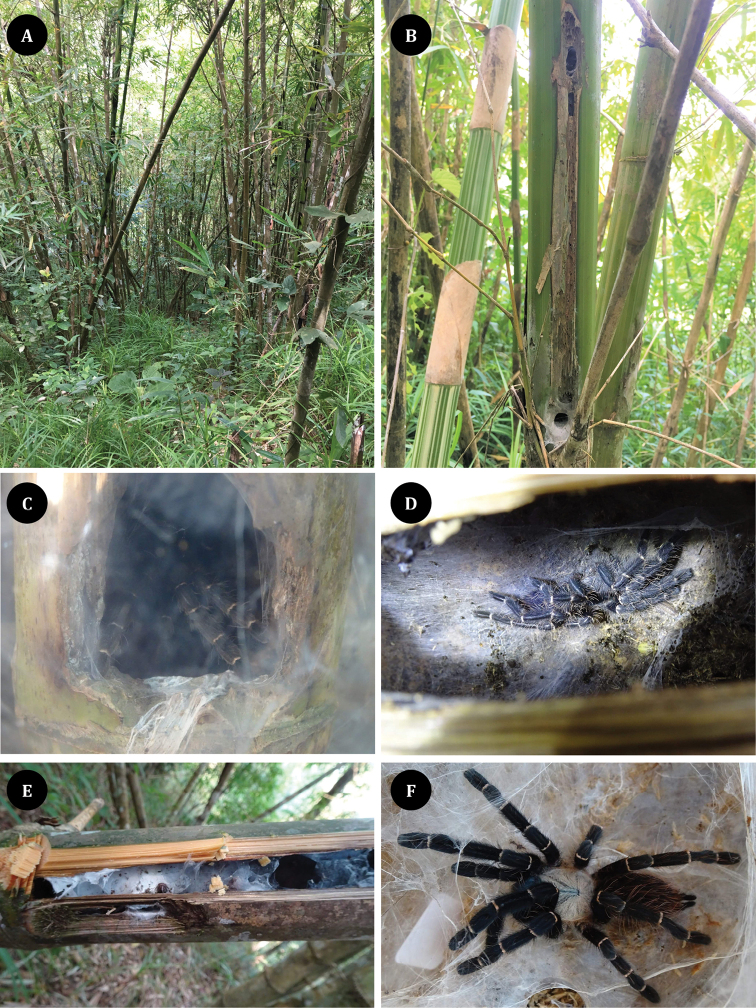
*Taksinusbambus* sp. nov. from Tak province, Thailand **A** biotope, bamboo forests in mountainous slope areas **B** tarantula habitat in bamboo culm with entrance hole (below) and secondary entrance (upper) **C, D** tarantula in bamboo culm **E** tarantula building silk tube retreats on the cover culm **F** paratype ♀, TAK3 *T.bambus* alive.

Figures 1 , 3 , 4 , 5A–E , 6 , 7 , 8

#### Type material.

Thailand • ***Holotype*** 1 ♂ (TAK1); Mae Tho, Mueang Tak district, Tak province. ***Paratype*** 2 ♀ (TAK 2–3 ♀); the same data as the holotype. Specimens were deposited at ENTOKKU (holotype TAK1 ♂ and paratype TAK2 ♀ ID: ENTOKKU TAK1–2) and THNHM (paratype TAK3 ♀ ID: THNHM-Ar-00005).

#### Diagnosis.

Same as for the genus.

#### Etymology.

The species name *bambus* refers to the species, which was discovered in a bamboo plantation and lives in Asian bamboo stalks.

#### Description.

Male TAK1 holotype (ENTOKKU): color in life: leg black, carapace brownish yellow. Total length (including chelicerae) 26.30 mm; cephalothorax 11.09 mm long, 7.62 mm wide, 4.40 mm high (caput); fovea 2.28 mm wide, straight, deep; cephalothorax brown, with a cover of short, whitish-yellow hairs dorsally, long whitish-yellow hairs on lateral margins; clypeus 0.23 mm; ocular tubercle 1.50 mm long, 2.59 mm wide. The anterior eye row slightly procurved and posterior row straight; eyes whitish, ALE oval and larger than the round AME; Eye sizes: AME, 0.45 mm; ALE, 0.88 mm; PLE, 0.48 mm; PME, 0.31 mm. Inter-eye distances: AME–AME, 0.62 mm; AME–ALE, 0.32 mm; AME–PME, 0.30 mm; ALE–ALE, 1.90 mm; ALE–PME, 0.34 mm; PME–PME, 1.40 mm; PME–PLE, 0.10 mm; PLE–PLE, 2.00 mm; and ALE–PLE, 0.32 mm. Chelicerae dark brown, 7.65 mm long, outer cheliceral face with short scopula edge with rows of orange-red setae, the lower surface of the outer cheliceral has five slightly curved plumose setae pads on the retrolateral chelicerae (Fig. 3A). Maxillae reddish brown, 6.06 mm long, 3.49 mm wide with >155 cuspules, covered with orange-red setae on the prolateral surface; stridulation organ consisting of stout thorn-like spines with 11 in two rows (7.4 mm from below suture) and six in one row (1.9 mm from above suture) on the prolateral maxillae (Fig. 3B). Labium brown, length 1.3 mm, width 2.0 mm, with >75 cuspules damaged and loss encompassing approximately 40% of the proximal edge (Fig. 3C). Sternum dark brown, covered with two hair types: strong dark and soft white; 6.41 mm long, 4.38 mm wide with two pairs of ovoid sigillae present near the lateral margins opposing coxa II and III. Sigilla: anterior pair absent; median pair 0.40 mm long, 0.25 mm wide, close to the sternal margin; posterior pair 0.79 mm long, 0.36 mm wide, 0.66 mm from the sternal margin. Abdomen 10.89 mm long, 7.71 wide, dark gray, black thickly hirsute laterally and ventrally. Legs: Pat, Tib, Met, and Tar dark brown. Length of legs and palpal segments are shown in Table 1, leg formula 1423. Spination: tibia II r 0–0–1 (apical), III r 0–0–2 (apical), metatarsus I v 0–0–1 (apical), II v 0–0–1 (apical), III d 0–0–1 (apical), v 0–0–1 (apical), IV d 0–0–1 (apical), v 0–0–3 (apical).

**Table 1.** Legs and palp measurements (in mm) of the holotype ♂ TAK1 *Taksinusbambus* sp. nov.

	I	II	III	IV	Palp
Fem	12.98	12.23	9.28	12.66	7.84
Pat	5.69	5.50	5.40	5.91	3.92
Tib	11.94	10.14	7.30	10.44	6.77
Met	9.32	7.45	8.35	11.07	–
Tar	6.43	5.37	4.95	5.80	3.48
Total	46.36	40.69	35.28	45.88	22.01

The male tibia I spur is present and lacks a single megaspine on the inside of the tibial apophyses (Fig. 4A–D). Scopulae on metatarsi and tarsi I through IV, undivided. Tar I–IV with two claws; spinnerets covered with dark longer and thinner hairs; Posterior lateral spinnerets with three segments, basal 2.3 mm, median 1.5 mm, digitiform apical 3.0 mm; lateral median spinnerets with one segment 1.25 mm. Pedipalps reddish brown, covered with longer and thinner hairs; tibia swollen; cymbium with two lobes of light brown shaggy scopulae; bulb and embolus 1.76 mm long, dark reddish brown; palpal bulb ellipsoid and partly concave, 1.60 mm long, 1.63 mm wide; embolus moderately curved, rounded apex, with single retrolateral keel (Fig. 5A–E).

Paratype ♀ TAK3: total length (including chelicerae) 30.82 mm; cephalothorax 13.43 mm long, 10.39 mm wide, 2.98 mm high (caput); fovea 1.28 mm wide, slightly procurved, deep; cephalothorax brown, covered with short whitish hairs dorsally, golden yellow to yellowish-brown long hairs on lateral margins (Fig. 6A); clypeus 0.13 high; ocular tubercle 1.70 mm long, 2.92 mm wide. Anterior eyes with long hairs in front of AME and mid-posterior PME area; anterior eye row slightly procurved and posterior row straight. Eye sizes: AME, 0.45 mm; ALE, 0.71 mm; PLE, 0.54 mm; PME, 0.37 mm. Inter-eye distances: AME–AME mm, 0.47; AME–ALE, 0.41 mm; AME–PME, 0.25 mm; ALE–ALE, 1.75 mm; ALE–PME, 0.54 mm; PME–PME, 1.27 mm; PME–PLE, 0.66 mm; PLE–PLE, 1.57 mm; and ALE–PLE, 0.39 mm. Chelicerae dark brown, 6.78 mm long, outer cheliceral face with short scopula margin with rows of orange-red setae, outer cheliceral on the lower surface with five slightly curved pads of plumose setae on the retrolateral chelicerae. Maxillae reddish brown, 3.83 mm long, 2.10 mm wide with >183 cuspules, covered with orange-red setae on the prolateral surface, labium brown, length 1.51 mm, width 2.09 mm, with >125 cuspules covering approximately 40% of the proximal edge (Fig. 6D). Sternum dark brown, covered with two types of hairs: strong dark and soft white; 5.89 mm long, 5.54 mm wide with two pairs of ovoid sigillae present near lateral margins opposite coxa II and III (Fig. 6B). Sigilla: anterior pair absent; median pair 0.35 mm long, 0.27 mm wide, close to the sternal margin; posterior pair 0.75 mm long, 0.32 mm wide, 0.72 mm from the sternal margin. Abdomen 15.58 mm long, 10.95 mm wide, dark gray and black thickly hirsute laterally and ventrally. Legs: Pat, Tib, Met, and Tar dark brown. Length of legs, palpal segments, and the comparative leg measurements are shown in Table 2, leg formula 4123. Spination: tibia palp r 0–0–1 (apical), p 0–0–1 (apical), r 0–0–2 (apical), I p 0–0–1 (apical), II p 0–0–1 (apical), r 0–0–1 (apical), III p 0–0–1 (apical), IV r 0–0–1 (apical), p 0–0–1 (apical), metatarsus I v 0–0–1 (apical), II v 0–0–1 (apical), III d 0–0–1 (apical), v 0–0–1 (apical), p 0–0–1 (apical), r 0–0–2 (apical), IV v 0–0–3 (apical). Scopulae on metatarsi and tarsi I through IV undivided. Tar I–IV with two claws; spinnerets covered with dark brown longer and thinner hairs; posterior lateral spinnerets with three segments, basal 2.03 mm, median 1.40 mm, digitiform apical 2.10 mm; lateral median spinnerets with one segment 1.21 mm. Spermathecae (Fig. 7F): paired and divided with fused in base, base 0.88 mm (left) and 1.00 mm (right) long, 1.10 mm (left) and 1.06 mm (right) wide; sclerotization heaviest apically, gradually decreasing basally.

**Table 2.** Legs and palp measurements (in mm) of paratype TAK3 ♀ *Taksinusbambus* sp. nov. from Thailand.

	I	II	III	IV	Palp
Fem	9.62	7.69	7.66	8.90	6.77
Pat	5.45	4.99	4.40	5.11	4.71
Tib	7.21	5.48	5.24	7.97	4.01
Met	5.76	4.67	5.63	8.10	–
Tar	4.20	4.21	3.84	4.57	4.81
Total	32.24	27.04	26.77	34.65	20.3

#### Description.

Paratype ♀ TAK2: dark brown, carapace brown. Total length (including chelicerae) 34.80 mm; cephalothorax 14.39 mm long, 11.57 mm wide, 3.16 mm high (caput); fovea 1.20 mm wide, straight, deep; cephalothorax brown, covered with short whitish hairs dorsally, golden yellow to yellowish-brown long hairs on lateral margins; clypeus 0.15 mm high; ocular tubercle 1.83 mm long, 2.70 mm wide. Anterior eyes with long hairs in front of AME and mid-posterior PME area; anterior eye row slightly procurved and posterior row straight. Eyes whitish, ALEs oval in shape, larger than the round AMEs. Eye sizes: AME, 0.44 mm; ALE, 0.69 mm; PLE, 0.59 mm; PME, 0.40 mm. Inter-eye distances: AME–AME, 0.37 mm; AME–ALE, 0.49 mm; AME–PME, 0.30 mm; ALE–ALE, 1.69 mm; ALE–PME, 0.68 mm; PME–PME, 1.20 mm; PME–PLE, 0.17 mm; PLE–PLE, 1.86 mm; and ALE–PLE, 0.50 mm. Chelicerae dark brown, 7.02 mm long, outer cheliceral face with short scopula margin with rows of orange-red setae; outer cheliceral on the lower surface with five slightly curved pad of plumose setae on the retrolateral chelicerae (Fig. 7B, C) with cheliceral needle form strikers (Fig. 7B, D). Maxillae reddish brown, 3.64 mm long, 2.21 mm wide with 149 cuspules, covered with orange-red setae on the prolateral surface, stridulation organ consisting of stout thorn-like spines with 10 in two rows (7.4 mm from below suture) and six in one row (3.0 mm from above suture) on the prolateral maxillae (Fig. 6C). Labium brown, length 2.29 mm, width 1.45 mm, with >7 cuspules damaged and lost. Sternum dark brown, covered with two types of hairs: strong dark and soft white; 6.22 mm long, 5.33 mm wide, with two pairs of ovoid sigillae present near the lateral margins opposite coxa II and III. Sigilla: anterior pair absent; median pair 0.51 mm long, 0.26 mm wide, close to the sternal margin; posterior pair 0.73 mm long, 0.32 mm wide, 0.45 mm from the sternal margin. Abdomen 18.72 mm long, 11.42 mm wide, dark gray and black thickly hirsute laterally and ventrally. Legs: Pat, Tib, Met, and Tar dark brown. Length of legs, palpal segments, and the comparative leg measurements are shown in Table 3, leg formula 4123. Spination: tibia palp r 0–0–1 (apical), p 0–0–1 (apical), r 0–0–2 (apical), I p 0–0–1 (apical), II p 0–0–1 (apical), r 0–0–1 (apical), III p 0–0–1 (apical), IV r 0–0–2 (apical), p 0–0–1 (apical), metatarsus I v 0–0–1 (apical), II v 0–0–1 (apical), III v 0–0–1 (apical), p 0–0–1 (apical), r 0–0–2 (apical), IV v 0–0–3 (apical). Scopulae on metatarsi and tarsi I through IV, undivided. Tar I–IV with two claws; spinnerets covered with dark longer and thinner hairs; posterior lateral spinnerets with three segments; basal 2.10 mm, median 1.36 mm, digitiform apical 2.19 mm; lateral median spinnerets with one segment 1.34 mm. Spermathecae (Fig. 7E): paired and divided with fused in base, base 0.90 mm (left) and 1.07 mm (right) long, 1.09 mm (left) and 1.17 mm (right) wide; sclerotization heaviest apically, gradually decreasing basally.

**Table 3.** Legs and palp measurements (in mm) of paratype TAK2 ♀ *Taksinusbambus* sp. nov. from Thailand.

	I	II	III	IV	Palp
Fem	9.33	8.46	7.08	9.67	6.59
Pat	5.07	4.04	3.6	4.58	3.39
Tib	6.66	6.37	5.33	8.25	4.23
Met	6.09	5.04	4.74	7.1	–
Tar	4.79	4.8	3.88	5.67	4.83
Total	31.94	28.71	24.63	35.27	–

#### Distribution and natural history.

Specimens were collected from villages surrounding Tak province at approximately 1,000 m elevation. The biotope consists of a mixed deciduous forest dominated by bamboo that is rarely disturbed by human activity (Fig. 8A). The new arboreal tarantula shows a surprising specialization in that it lives in the stalks of Asian bamboo (*Gigantochloa* sp.) (Fig. 8B–E). All specimens were collected from bamboo internodes in mature culms, having nest entrances approximately 2–3 cm within a silk-lined tubular burrow at the entrance located in the branch stub or at the middle of the bamboo culms. Some specimens had a secondary entrance without silk at the hole (Fig. 8B). Tarantulas do not bore bamboo stems; instead, they depend on the assistance of other animals. Bamboo is attacked by numerous animals, the most common of which are insects from the orders Coleoptera, Lepidoptera, and Diptera (Varma and Sajeev 2015). Furthermore, we hypothesized that the tarantula might occupy the empty nest of insects, such as the bamboo-nesting carpenter bee *Xylocopa*, which creates a large hole. All the tarantulas living in the bamboo culms build silken retreat tubes that cover the stem cavity (Fig. 8C–E).

## ﻿Discussion and conclusion

**Figures 9.**
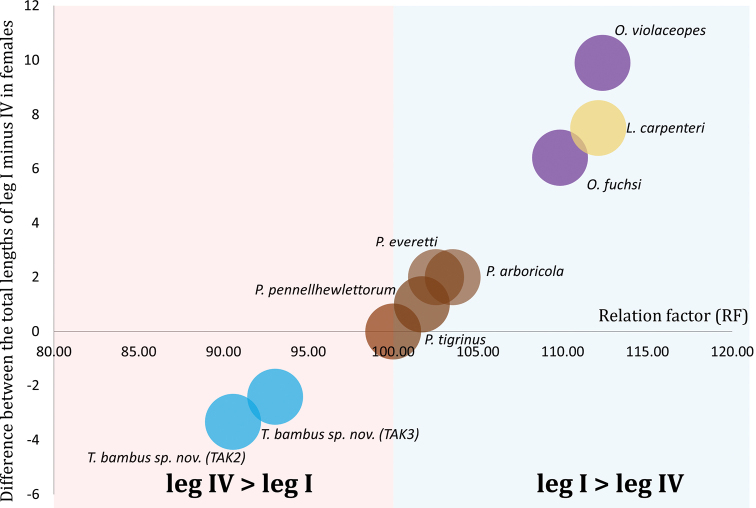
Scatter plot illustrating the difference between the total leg lengths I minus IV and the relation factor (RF) of arboreal Ornithoctoninae. The red area contains data indicating that species have a longer leg IV (leg formula = 4123), whereas the blue area has data indicating that species have a longer leg I (leg formula = 1423).

Recently, Gabriel and Sherwood (2019) revised some arboreal Ornithoctoninae and proposed various stable morphological features that enable generic-level delineation, including the presence of the embolus rising parallel and sloping to a point at the tip, which distinguishes the genus *Omothymus* from *Phormingochilus*; embolus apical swelling in *Lampropelma* and the difference between the total lengths of leg I and IV (± 2–3 mm difference in *Phormingochilus*, ± 5 mm in *Lampropelma* and ± 10 mm in *Omothymus*) in all cases, leg I is longer than leg IV. Von Wirth and Striffler (2005) proposed a similar concept for terrestrial Ornithoctoninae called leg relation factor (RF), although Leg RF is a mathematical formula that expresses the relationship as a decimal value. We examined both methodologies and included *Taksinus* gen. nov. in our analysis, using leg measurements from original species descriptions or study type material obtained from the World Spider Catalog (2021), as shown in Table 4. The result indicated that *Taksinus* gen. nov. had the minimum difference between the total length of leg I minus IV = −3.33 to −2.41 (RF = 90.56–93.04), whereas *O.violaceopes* had the maximum height at +9.9 (RF = 112.3). According to Gabriel and Sherwood’s criterion, *Taksinus* gen. nov. and *Phormingochilus* share a leg measuring range ± 2–3 mm, indicating that they belong to the same genus, although their RF are clearly dissimilar. After evaluating *Taksinus* gen. nov., we conclude that the range based on the generic number of female variations in leg I and IV length cannot be used to classify all arboreal Ornithoctoninae. Furthermore, we plot charts to show the value distribution for each species by comparing the total lengths of leg I–IV and the RF (Fig. 9). This study indicated that species on the left (red area) have a longer leg IV than their leg I (leg formula = 4123), whereas species on the right (blue area) have a longer leg I than their leg IV (leg formula = 1423). The comparative approach used in this study revealed that *Taksinus* gen. nov. appeared on the left side, with legs varying by −3.33 to −2.41 (RF = 90.56–93.04), whereas *Lampropelma* and *Omothymus* appeared on the right side with legs varying by +6.4 to +9.9 of the total length of leg I minus IV and RF greater than 100 (RF = 109–112.3). The variation in the length difference between *Phormingochilus* legs I and IV is centered on the charts, ranging from 0 to 2 (RF = 100–103.5). A visual leg length comparison of arboreal Ornithoctoninae, using both methodologies is indicated in Fig. 9. Gabriel and Sherwood (2019) employed a generic number that enables rapid assessment for a defined difference of *Omothymus*, *Phormingochilus*, and *Lampropelma*, but whilst useful for those genera, *Taksinus* gen. nov. cannot be defined using this method. In contrast, the RF can be used to calculate a decimal value for leg proportions. For instance, a value greater than 100, such as *O.violaceopes* 112.3, indicates that this species has short hind legs, whereas a value less than 100 indicates that this species has long hind legs. The RF, however, was developed primarily to diagnose species rather than for higher-level delineation and needs to be further evaluated before it could be reliably used at the genus level.

**Table 4.** Comparative leg measurements of legs I and IV (female) and relation factor (RF) of arboreal Ornithoctoninae from original species descriptions or study type material.

Species (Female)	Reference from World Spider Catalog (2021) and Type material	Leg I (mm)	Leg IV (mm)	Leg formula	The total lengths of leg I minus IV in females	Relation factor (RF)
*T.bambusinus* sp. nov.	(paratype ENTOKKU TAK2)	31.94	35.27	4123	-3.33	90.56
*T.bambusinus* sp. nov.	(paratype ENTOKKU TAK3)	32.24	34.65	4123	-2.41	93.04
*P.tigrinus* Pocock, 1895	personal examination of this publication’s reviewers (holotype BMNH 1894.6.27.1)	50.20	50.20	(1=4)23	0	100
*P.tigrinus* Pocock, 1895	personal examination of this publication’s reviewers (holotype BMNH 1894.6.27.1)	54	53	1423	1	101.89
*P.pennellhewlettorum* Smith & Jacobi, 2015	*Phormingochiluspennellhewlettorum* Smith & Jacobi, 2015: 38, figs 24–40, 44–49 (holotype)	60	59	1423	1	101.7
*P.everetti* Pocock, 1895	*Phormingochiluseverettii* Pocock, 1895: 180, pl. 10, fig. 4 (Df). (holotype BMNH 88.122)	81	79	1423	2	102.5
*P.arboricola* (Schmidt & Barensteiner, 2015)	*Lampropelmanigerrimumarboricola* (Schmidt & Barensteiner, 2015): 5, figs 1–4(f). (holotype)	59	57	1423	2	103.5
*O.fuchsi* (Strand, 1906)	*Phormingochilusfuchsi* examined by Smith, 1994: 22, fig. 16(f). (holotype MWNH 319)	71.4	65	1423	6.4	109.8
*L.carpenteri* (Smith & Jacobi, 2015)	*Phormingochiluscarpenteri* Smith & Jacobi, 2015: 34, figs 10–16(Df). (holotype BMNH)	69.5	62	1423	7.5	112.1
*O.violaceopes* (Abraham, 1924)	*Lampropelmaviolaceopedes* (Abraham, 1924): 1108, pl. 5, figs 19–24 (holotype BMNH 1924.27.19.1.37)	90.1	80.2	1423	9.9	112.3

Evaluation of the geographic distributions of Asia arboreal tarantula currently identified within the Ornithoctoninae subfamily—*Lampropelma*, *Omothymus*, and *Phormingochilus*, and *Taksinus* gen. nov. (Fig. 2) provided interesting data. We classified *T.bambus* in a new genus rather than *Omothymus*, which occurs in Sumatra and peninsular Malaysia due to the consistency in the morphological features of *T.bambus* sp. nov., which have a rounded, gentle curve and reduced ridges on the embolus (*Taksinus* gen. nov. Fig. 5A, B; *Omothymus* Fig. 5F–J; see Gabriel and Sherwood 2019: 139, figs 1–5), and embolus is short compared to palpal bulb length (1:1). *T.bambus* sp. nov. is closely related to *Phormingochilus* based on palpal bulb morphology (see Gabriel and Sherwood 2019: 142, figs 9–13), with the palpal bulb’s rounded apex in apical view. Based on Gabriel and Sherwood (2019), *T.bambus* sp. nov. is similar to *Phormingochilus* in terms of the total lengths of leg I and IV in females ±2.41–3.33 mm (±2–3 in *Phormingochilus*; see Gabriel and Sherwood 2019); they differ when the leg formula is calculated as the length of leg I minus that of leg IV (*Taksinus* gen. nov. are −2 to −3 while *Phormingochilus* indicated that leg I is longer with +2 to +3 of the value). Furthermore, *Phormingochilus* is found solely on Borneo Island, posing a considerable geographic barrier to northern Thailand (Fig. 2). Conclusively, our findings indicated that *Taksinus* gen. nov. showed significant differences in its morphology and comparative leg measurements compared to other Ornithoctoninae arboreal tarantulas. Nonetheless, future researchers should investigate and characterize these genera using molecular phylogenetic approaches.

## Supplementary Material

XML Treatment for Taksinus

XML Treatment for Taksinus bambus
